# Tension gastrothorax in late-onset congenital diaphragmatic hernia, a rare but life-threatening condition: A case report

**DOI:** 10.1097/MD.0000000000024815

**Published:** 2021-02-19

**Authors:** In-Hag Song

**Affiliations:** Department of Thoracic and Cardiovascular Surgery, Soonchunhyang University Cheonan Hospital, Chenan-si, South Korea.

**Keywords:** congenital diaphragmatic hernia, life-threatening condition, tension gastrothorax

## Abstract

**Rationale:**

Tension gastrothorax is a serious condition that can cause acute respiratory failure, which is mostly related to congenital diaphragmatic hernia (CDH) in pediatric cases. It is uncommon in late-onset CDH patients, and is difficult to diagnose due to atypical presentation. It is often misdiagnosed as tension pneumothorax or pleural effusion, leading to delayed treatment and potentially fatal outcome. In this study, we are reporting our experience of diagnosis and treatment of tension gastrothorax in a late-onset CDH patient.

**Patient concerns:**

A 2-year old boy presented to this hospital with severe dyspnea and abdominal pain that suddenly occurred while taking a bath.

**Diagnosis:**

Based on radiological findings we diagnosed tension gastrothorax.

**Interventions:**

Hernia reduction and diaphragmatic defect repair were performed under thoracotomy.

**Outcomes:**

After the operation, the patient's clinical symptoms and imaging findings improved. At 1-year postoperative follow up, the patient was well with normal chest x-ray findings.

**Lessons:**

Tension gastrothorax in late-onset CDH is a life-threatening condition that requires rapid diagnosis and treatment. When the diagnosis is unclear by chest x-ray, chest computed tomography should be performed to confirm the diagnosis. A nasogastric tube should be inserted whenever possible for diagnosis and gastric decompression. Although laparotomy is the most preferred approach, we recommend that surgeons consider taking a thoracotomy approach in unstable patients that cannot undergo gastric decompression before operation.

## 1 Introduction

Tension gastrothorax can occur in patients with congenital or acquired diaphragmatic defect.^[1–3]^ It is caused by the stomach herniating into the thoracic cavity through the diaphragmatic hernia, causing significant distention of the stomach by gas and/or fluid. This in turn leads to mediastinum shifting, leading to sudden serious acute respiratory failure that can potentially be fatal.^[2,3]^ Most pediatric tension gastrothorax is caused by congenital posterolateral defect in the diaphragm that leads to stomach hernia.^[1–3]^ It often has atypical presentation and is commonly mistaken for tension pneumothorax or pleural effusion on chest x-ray, leading to incorrect management such as thoracostomy that delays appropriate management and potentially leads to fatal outcome.^[1–4]^ Therefore, rapid and accurate diagnosis and treatment is crucial for management of this condition.

## 2 Case report

**Figure 1:**
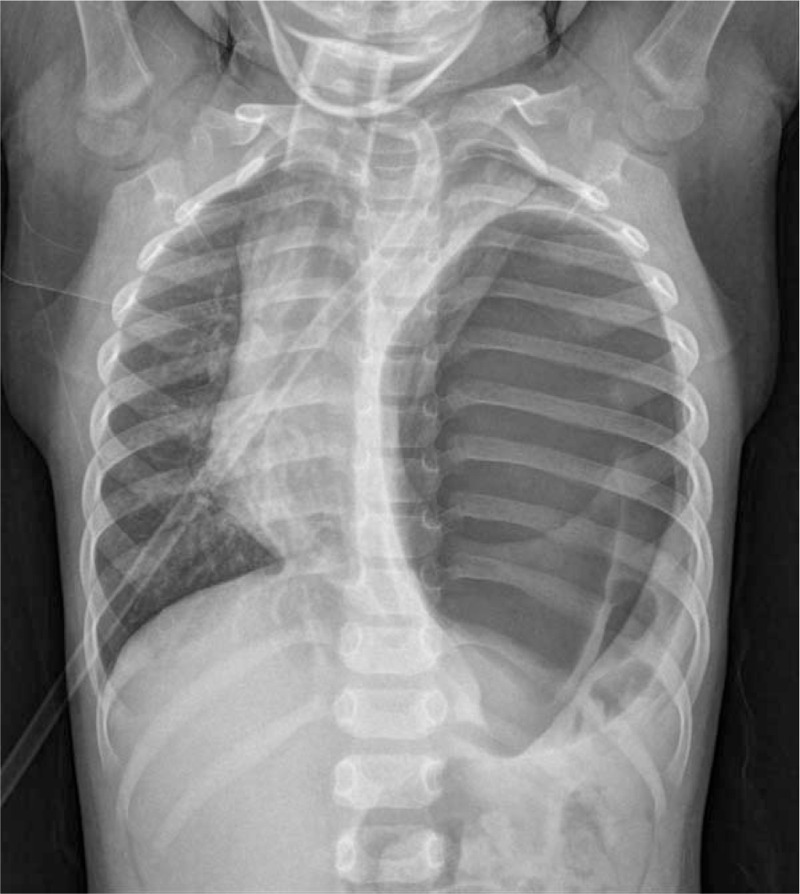
Chest x-ray showing air shadowing filling the entire left thorax and mediastinum shifting.

A 2-year old boy without significant past medical history presented to the pediatric emergency department of this hospital following extreme irritability, crying, abdominal pain, and shortness of breath that occurred during bath time. Initial blood pressure was 90/50 mm Hg, pulse rate and respiratory rate were 161/min and 50/min respectively, temperature was 37.3°C and oxygen saturation was 88% to 91% on room air, so he was given oxygen by mask. He had wheezing on the right side of the chest with reduced breath sounds on the left side. The white blood cell count was elevated at 38,450/uL, and his chest x-ray showed air shadowing in the whole left thorax and mediastinal shift (Fig. 1).

**Figure 2:**
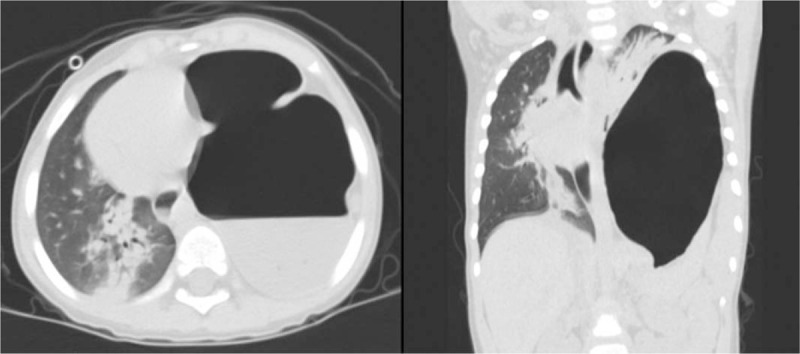
Chest computed tomography (CT) scan showing the stomach herniated through the diaphragmatic defect with significant distension with fluid and air, leading to total collapse of the left lung and shifting of the mediastinum shifting, as well as right-sided pneumonia.

The pediatric emergency team suspected a large bulla or tension pneumothorax in the left lung, so the patient was referred to our department for opinion. Based on the chest x-ray, we did not feel that it was a bulla or pneumothorax so the patient underwent chest computed tomography (CT) for more accurate diagnosis. The chest CT showed a herniated stomach that was distended with large amount of food and gas, leading to significant shifting of the heart and lung to the right side, as well as right-sided pneumonia (Fig. 2). Based on these findings we diagnosed tension gastrothorax and nasogastric tube (NGT) was inserted for gastric decompression, but the patient was irritable and did not tolerate it so it was soon pulled out.

The oxygen saturation level of the patient could not be maintained despite high flow oxygen through a mask and the patient had high fever with ipsilateral pneumonia. It was deemed unsafe for the patient to have NGT reinsertion while awake, so a decision was made to put the patient under general anesthesia for NGT insertion followed by operation. However, the patient had cardiac arrest immediately after starting general anesthesia in the operating room. Although the circulation returned after resuscitation, the vital signs were unstable. Considering the risk of another cardiac arrest during NGT insertion, we decided to proceed to operation without reinserting the NGT. As gastric decompression could not be achieved, we thought that laparotomy approach would be difficult so proceeded to thoracotomy approach.

Posterolateral thoracotomy showed a significantly distended stomach that filled the entire thoracic cavity. In order to aid the recovery of the cardiorespiratory function, there was a need for immediate gastric decompression to relieve the pressure on the heart and lungs from the distended stomach. The stomach was; therefore, deliberately perforated to suction the gas and food contents filling the stomach, to relieve the pressure on the lungs and the heart. Once patient vitals were stabilized, the stomach perforation site was closed and the herniated stomach was examined which did not reveal ischemic changes or other abnormalities.

The diaphragmatic defect was found on the posterolateral side. The stomach that had herniated through the defect was reduced, and as the defect was not very large, we decided that patch reconstruction was not necessary. The defect was treated by primary repair.

**Figure 3:**
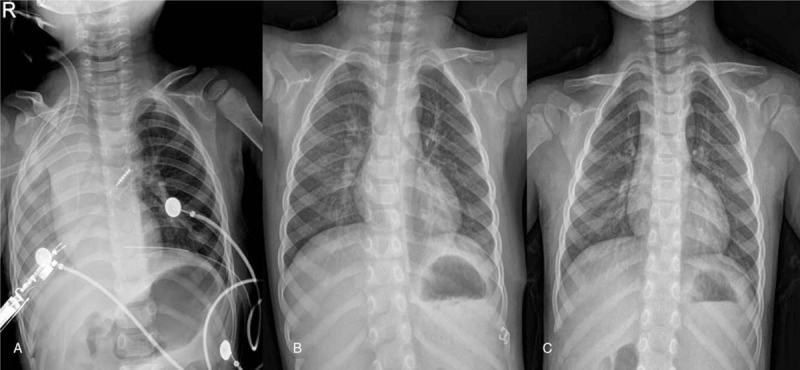
Chest x-rays from immediately after surgery (A), time of discharge (B) and 1 yr follow up (C). The diaphragm is now normal in shape, and normal location of the stomach gas.

Postoperative chest x-ray showed full expansion of the left lung, with normal diaphragm. There was normal stomach gas in the intraperitoneal region (Fig. 3A). The chest tube was removed on day 3, and the patient was discharged on day 8 when the right-sided pneumonia had improved (Fig. 3B). At 1-year postoperative follow up, the patient was well with normal chest x-ray findings (Fig. 3C).

This case report was approved by the Institutional Review Board of Soonchunhyang University Hospital (SCHCA 2020-07-010). The patient has provided informed consent for publication of this case.

## 3 Discussion

Tension gastrothorax is caused by rapid increase in the abdominal pressure, leading to herniation of the stomach through the pre-existing diaphragmatic defect. This then leads to abnormal distortion of the gastro-esophageal junction at the diaphragmatic defect level, causing gastric outlet obstruction, creating a 1-way valve to cause rapid distention of the stomach with gas and/or fluids.^[2]^ Tension gastrothorax causes acute respiratory failure and gastrointestinal obstruction, and shifting of the mediastinum that compresses the heart and major blood vessels leading to shock and cardiac arrest in some cases.^[2,3]^

As mentioned above, tension gastrothorax is caused by significant distension of the herniated stomach into the thoracic cavity through a congenital or acquired (traumatic or iatrogenic) diaphragmatic defect. When it occurs in pediatrics, the right side is buttressed by the liver, so most cases are caused by a hernia of the stomach through a congenital defect on the posterolateral side (Bochdalek) of the left diaphragm.^[1–3]^

The majority of congenital diaphragmatic hernia (CDH) (50%–90%) is diagnosed on obstetric ultrasound. However, approximately 5% are found after the neonatal period.^[5]^ When the diaphragmatic hernia is small, it is protected by the liver on the right, and the spleen on the left, so often there are no symptoms for several months to years of life and patients often have normal chest x-ray.^[5–7]^ Late-onset CDH is less common, and can present with various symptoms, such as cough, dyspnea, chest pain, frequent respiratory infections, abdominal pain, diarrhea, and vomiting. In severe cases, patients can present with serious respiratory failure or cardiopulmonary failure. This makes diagnosis of CDH difficult and it is often mistaken for other conditions.^[2,5–7]^

The patient in this report did not have CDH diagnosed during obstetric ultrasound, and based on the history of no symptoms since birth we suspected a small diaphragmatic defect, which was later confirmed during operation. Since the defect was small, it was likely that the hernia was protected by the spleen so that the organs had not herniated through the defect. It is possible that a certain event where the abdominal pressure was suddenly increased caused the stomach to herniate through the defect in the diaphragm.

Tension gastrothorax is a potentially life-threatening condition, so must be diagnosed immediately.^[2,3,6]^ The initial investigation is chest x-ray^[3,6]^ which can show the following: left thoracic structure filled with air, with or without fluid level, concomitant collapsed ipsilateral lung. The mediastinum is shifted to the right, and stomach gas is not seen in left upper abdomen. The left hemidiaphragm is also frequently not seen, which is an important differentiation with tension pneumothorax.^[2,3]^

Nevertheless, tension gastrothorax is often mistaken for tension pneumothorax or pleural effusion, leading to inappropriate thoracostomy.^[3–5]^ This leads to delayed management, and inappropriate insertion of the chest tube can cause gastric perforation that can cause gastric contents leakage to the thoracic cavity.^[3,6,7]^ Hence it is important that the chest x-rays should be carefully observed, and if the diagnosis is unclear with chest x-ray alone, CT scan is recommended for accurate diagnosis and prevention of inappropriate invasive procedures, such as thoracostomy.^[6]^

The patient in this report was referred to our department for suspected tension pneumothorax or large bulla, based on large air shadow and mediastinal shifting on his chest x-ray, and symptoms of dyspnea. However, after careful observation of the chest x-ray we did not believe that it was pneumothorax or bulla, and proceeded to CT imaging of the thorax that confirmed tension gastrothorax. This prevented inappropriate procedures and also allowed for the diagnosis of right-sided pneumonia.

NGT insertion is a procedure that is used to confirm tension gastrothorax and provide initial treatment. The presence of the NGT tip in the thoracic cavity on chest x-ray confirms the diagnosis of gastrothorax, and decompresses the distended stomach to improve symptoms.^[2–7]^ This was attempted in our patient, but the patient was very irritable and could not tolerate the NGT so it was pulled out immediately after insertion. The patient was a young child, and his condition rapidly deteriorated. Given his irritability and unstable condition it was decided that repeat NGT insertion would be difficult. We were concerned that re-attempting NGT insertion with pneumonia in the opposite lung might cause worsening of the tension, or aspiration pneumonia from vomiting.

Therefore we planned to put the patient under general anesthesia to undergo gastric decompression by NGT insertion, followed by surgery. But as mentioned previously, the patient immediately developed cardiac arrest following general anesthesia and was resuscitated. Although spontaneous circulation was achieved following resuscitation, the vital signs were still unstable. We did not want to risk another cardiac arrest by reattempting NGT insertion so we proceeded with operation without NGT.

Many authors recommend needle thoracentesis under chest x-ray guidance to decompress the stomach before operation.^[2,3]^ However, considering the clinical status of the patient before operation, we decided that preoperative needle thoracentesis was difficult.

Laparotomy approach is an often preferred surgical method^[2,3,5–7]^ as it allows rapid reduction of the herniated organs, examination of the intraabdominal organs, and easier repair of the diaphragmatic hernia.^[2]^

In this case, we could not achieve gastric decompression before operation. As the patient had to be resuscitated in the operating room following cardiac arrest and had very unstable vitals following resuscitation, we decided to take thoracotomy approach to achieve rapid gastric decompression and examination of the lungs and the thoracic cavity within the operative field. The outcome was a safe and relatively easy operation.

Based on our experience, we suggest that surgeons consider using thoracotomy approach in patients who cannot undergo gastric decompression before operation. Thoracotomy could also be considered in patients who have stomach perforation complication and subsequent leakage of stomach contents from incorrect thoracentesis based on incorrect diagnosis of tension pneumothorax or pleural effusion.

In conclusion, tension gastrothorax in late-onset CDH patients is uncommon and often has atypical presentation, making it difficult to differentiate with other conditions. However, it is a life-threatening condition that requires rapid diagnosis and treatment. Chest x-ray images must be observed closely and when the diagnosis is unclear, chest CT should be done to confirm the diagnosis. If possible, insertion of NGT provides both diagnosis and stomach decompression. Although laparotomy is the most preferred approach, we recommend that surgeons consider taking a thoracotomy approach in unstable patients that cannot undergo gastric decompression before operation.

## Author contributions

**Conceptualization:** In-Hag Song.

**Data curation:** In-Hag Song.

**Formal analysis:** In-Hag Song.

**Investigation:** In-Hag Song.

**Writing – original draft:** In-Hag Song.

**Writing – review & editing:** In-Hag Song.
